# Advancing our understanding of HIV co-infections and neurological disease using the humanized mouse

**DOI:** 10.1186/s12977-021-00559-z

**Published:** 2021-06-16

**Authors:** Janice J. Endsley, Matthew B. Huante, Kubra F. Naqvi, Benjamin B. Gelman, Mark A. Endsley

**Affiliations:** 1 Department of Microbiology and Immunology, University of Texas Medical Branch, Galveston, TX 77555 USA; 2 Department of Pathology, University of Texas Medical Branch, Galveston, TX 77555 USA

**Keywords:** Humanized mouse, Human immunodeficiency virus, *Mycobacterium tuberculosis*, *Neisseria gonorrhoeae*, Hepatitis C virus, Hepatitis B virus, Small animal model, Co-infections

## Abstract

Humanized mice have become an important workhorse model for HIV research. Advances that enabled development of a human immune system in immune deficient mouse strains have aided new basic research in HIV pathogenesis and immune dysfunction. The small animal features facilitate development of clinical interventions that are difficult to study in clinical cohorts, and avoid the high cost and regulatory burdens of using non-human primates. The model also overcomes the host restriction of HIV for human immune cells which limits discovery and translational research related to important co-infections of people living with HIV. In this review we emphasize recent advances in modeling bacterial and viral co-infections in the setting of HIV in humanized mice, especially neurological disease, and *Mycobacterium tuberculosis* and HIV co-infections. Applications of current and future co-infection models to address important clinical and research questions are further discussed. 
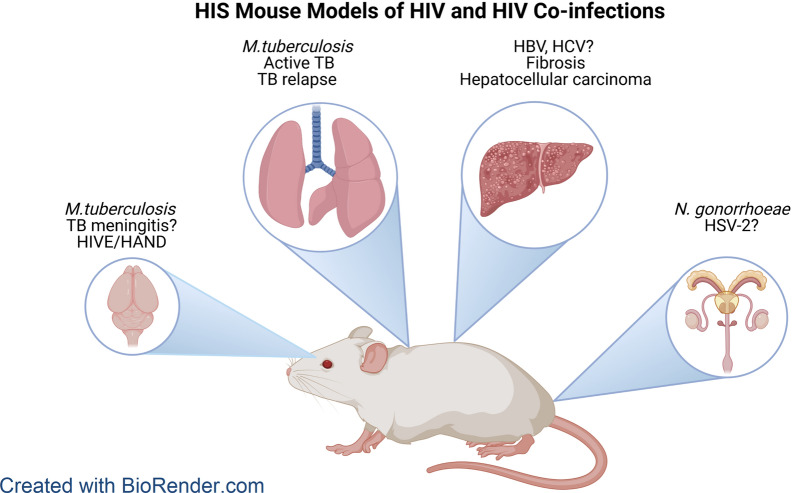

## Introduction

New and chronic infections with human immunodeficiency virus (HIV) remain a top global health concern 40 years into the pandemic. Approximately 39 million people are chronically infected with HIV, and 1.7 million become newly infected each year [1]. Development of antiretroviral therapy (ART) has transformed HIV infection from a fatal disease into a medically manageable chronic condition. Significant progress in providing access to HIV testing and ART has also greatly increased global awareness of HIV status (~ 81%) and ART access (~ 67%) in people with HIV (PWH) [1]. Access to ART has dramatically reduced incidence of several opportunistic infections (e.g. *Pneumocystis pneumonia*, Cytomegalovirus) that are important causes of death in people with low CD4^+^T cell count due to progression to Acquired Immune Deficiency Syndrome (AIDS) [2].

Among virally suppressed patients, a variety of co-morbidities and co-infections still produce clinically significant morbidity and mortality. A considerable proportion of the 39 million PWH, including those who are virally suppressed on ART, still suffer from HIV-associated conditions (e.g. advanced aging, hypertension) that can reduce lifespan and quality of life [3]. In 2019, deaths due to HIV-related illness were estimated at 700,000 [1]. Tuberculosis (TB), caused by infection with *Mycobacterium tuberculosis* (*Mtb*), is a leading cause of death in PWH, accounting for one in three AIDS-related deaths [4]. Sexually transmitted diseases such as *Neisseria gonorrhoeae*, *Chlamydia trachomatis,* and Herpes simplex virus 2 have a significantly higher prevalence in HIV-infected populations [5]. Co-infection with hepatitis C virus (HCV) and hepatitis B virus (HBV) are emerging as important factors contributing to increased rates and accelerated course of hepatocellular carcinoma in PWH [6, 7]. Development of prophylactic and therapeutic agents for these important co-infections of PWH has been limited due to the paucity of animal models that can support HIV replication. Non-human primate models that employ SIV can effectively model many important aspects of several diseases, including *Mtb*. However, the cost, regulatory issues, and length of natural infections in these models have long presented serious challenges to timely progress.

The human immune system (HIS) mouse is a standard system for in vivo studies of HIV resulting from over 20 years of research in variations of the model [see reviews [8, 9]]. The lower cost and ease of use of the small animal along with susceptibility to infection with human clinical isolates offer several advantages for basic and translational research. Research in HIS mice has significantly expanded our understanding of HIV including pathogenesis, mucosal transmission, latency and neurological manifestations. It has also emerged as a valuable translational platform for drug and vaccine development [10–17]. More recently the HIS mouse has been further adapted to facilitate study of co-infections including *Mycobacterium tuberculosis* and *Neisseria Gonorrhoeae* [18–20]. Recent adaptations of humanized mice that support development of human hepatocytes, and thus infection with HBV and HCV, are paving the way for HIS models of HIV co-infections with hepatitis viruses [21, 22]. Here we review how HIS models can advance our understanding of HIV neurological disease and important bacterial and viral co-infections. We further discuss the potential advantages and limitations of HIS models, and suggest applications that could advance our general understanding of these diseases.

## Humanized mouse models of HIV and TB

### The HIV and TB syndemic: need for co-infection models

TB and HIV infections have been the leading two causes of death due to infectious disease over many years prior to the COVID-19 pandemic. The two infections are considered a co-pandemic due to many factors including microbial synergy and overlapping epidemiology as we recently reviewed [23]. TB is the number one cause of death in people with HIV/AIDS because HIV infection: (1) increases susceptibility to *Mtb* infection and reactivation; (2) accelerates development of *Mtb* drug resistance; (3) compromises TB diagnostics; (4) is associated with drug interactions when combining TB chemotherapy and ART; and (5) causes an aggressive course of TB disease [24–27]. An increased risk of TB precedes CD4^+^ T cell depletion and can even continue following restoration of T cells by ART (see reviews [25, 28, 29]). The basis for this risk in virally suppressed persons is poorly understood. Speculatively, these outcomes could be driven by incomplete suppression in tissues with poor drug penetrance or immune disturbance due to chronic and systemic inflammation. Active tuberculosis can further promote HIV pathogenesis by increasing pulmonary virus replication, while latent TB is a serious risk factor for development of immune reconstitution inflammatory syndrome (IRIS) when ART is initiated [30]. Treatment of co-infected persons is challenging due to the aggressive course of both individual diseases, and often is further complicated by drug interaction and malabsorption when combining ART and TB chemotherapy [31].

Addressing these clinical challenges of the HIV and TB co-syndemic requires a much greater understanding of co-infection pathophysiology. Models that permit evidence-based pathogenesis studies are needed to inform development and implementation of countermeasures to complement ART and antimycobacterial therapies in co-infected people. To address this gap, our research group has developed two novel models of HIV/TB co-infection in HIS mice. As described below, these models of active co-infection and TB relapse offer systems to investigate mechanisms of microbial synergy and test novel interventions to treat co-infection.

### Active Mtb and HIV co-infection in the HIS mouse

Disease models that use standard mouse strains are indispensable in the TB research field for basic discovery and development of preventives and therapeutics. Depletion of CD4^+^T cells in vivo can simulate the effect of severe immune compromise, characteristic of AIDS in murine TB models [32]. The restriction of HIV for human host cells, however, has posed a continual challenge for development of systems to understand CD4^+^T cell loss-independent mechanisms of dual infection. The development of the HIS mouse as a small animal model of TB [33] was an important step that enabled application of humanized mice to understand co-infection. HIS mice, generated using the NOD SCID gamma (NSG) mouse and the bone marrow, liver, and thymus (BLT) method, were shown to develop a progressive, disseminating *Mtb* infection that produced caseous granulomatous inflammation consistent with human disease [33]. Lesions in the HIS model displayed typical gross pathology of *Mtb*-driven inflammation including organization of human CD3^+^T cells in the periphery of *Mtb* granulomas adjacent to human macrophages positive for acid fast bacilli (AFB). A human cellular immune response to *Mtb* infection was also demonstrated in the lung and periphery of infected HIS mice including activation of human effector cytokines (e.g. TNF-α and IFN-γ) that have defined roles in host defense and maintenance of latency. In two separate reports, HIS BLT mice that were infected with *Mycobacterium bovis* (*M. bovis*) BCG (vaccine strain) also were shown to mount an adaptive human immune response and develop lung lesions populated by human T cells and macrophages [34, 35]. Development of necrotic TB lesions, a hallmark of human TB pathology, is only observed to date in BLT mice infected with *Mtb,* and not BCG [33, 34]. These early studies demonstrated the potential to simulate important features of human pulmonary TB in a system replete with human immune cells and thus amenable to HIV infection.

An acute HIV infection model generated with BLT HIS mice was subsequently used as the platform to develop a model of *Mtb* co-infection [19]. HIV infection was established by intravenous injection 3 weeks prior to pulmonary *Mtb* challenge to simulate the progression of TB in the setting of active viral replication in a human immune system. Outcomes in this model reproduced several endpoints observed in co-infected persons including increased mycobacterial proliferation, disruption of organized granulomatous lesions including TB pneumonia, and development of exacerbated pro-inflammatory immune responses. Similar to observations in the rhesus macaque model of SIV/TB co-infection [36] and lung of a co-infected human lung specimen [19], HIV + cells are observed localized to *Mtb* lesions in the lungs of HIS mice [19]. Interestingly, the increased pro-inflammatory lung response and localization of HIV to *Mtb*-driven sites of inflammation was shown to precede the increase in lung bacterial load.

Although further investigations are needed, these results in an experimentally controlled setting suggest that recruitment or proliferation of HIV^+^ cells at the site of *Mtb* infection could change the local immune response to the advantage of *Mtb* pathogenesis. Similarly, the inflammatory host response to *Mtb* could provide a microenvironment advantageous for replication of HIV. This model sets the stage for carefully controlled experiments to address these and other important questions relevant to understanding co-infection pathophysiology.

### Model of TB relapse in the setting of HIV

A majority of the 10.4 million new TB cases that occur annually are in those who have access to TB drug therapy through public health programs [37]. Clinical cure is observed in most subjects following the standard combination drug therapy. A range of incomplete cures is also commonly observed and can include latent as well as active infections [38–40]. Relapse of TB in those with incomplete cures can occur immediately following treatment and most often is observed within the first year as reviewed [41]. The recurrence of TB due to relapse is strongly associated with the HIV pandemic and plays an important role in the global TB burden [42, 43]. In addition to having a greater risk for new *Mtb* infections, PWH are more likely to experience reactivation of latent TB, have greater rates of TB chemotherapy failure, and experience increased rates of recurrence after treatment [25, 44, 45]. Recurrent TB is also strongly associated with the development of multi-drug resistant (MDR)-TB or extensively drug resistant (XDR)-TB, which represent a global health crisis [4, 46, 47].

The immune basis for TB recurrence or relapse in those with HIV is poorly understood and difficult to investigate in affected human populations. The loss of both numbers and function of CD4^+^T cells is linked to latent TB reactivation, treatment failure, or relapse upon drug therapy completion (see reviews [48, 49]. An increased risk for TB relapse persists in PWH despite recovery of CD4^+^T cell numbers and viral suppression following ART [50, 51]. The natural development of latent TB infection (LTBI) in the absence of drug therapy occurs in NHP and can be experimentally reactivated with simian immunodeficiency virus (SIV) [36, 52]. The basis for SIV-mediated reactivation of LTBI has been shown to be associated with CD4^+^T cell loss-dependent and-independent mechanisms in different NHP species [52, 53]. To date, an NHP model of SIV-mediated TB relapse following drug treatment has not been described and immune events contributing to relapse are thus even less understood than latency reactivation.

BLT mice generated with an NSG background were used to develop a HIS mouse model of TB relapse following development of paucibacillary TB due to anti-mycobacterial drug therapy [18]. In these studies, animals were infected with *Mtb* and treated with the TB drugs rifampicin (RIF) and isoniazid (INH) to reproduce a Cornell-like model of latency in HIS BLT mice [18] similar to that established in mouse strains that are not susceptible to HIV [54, 55]. Infection of HIS mice with HIV after establishment of paucibacillary (non-culturable) *Mtb* infection with RIF/INH promoted an accelerated rate of regrowth in lung and a site of dissemination (liver). These results are consistent with the increased rates of TB relapse that occur following evidence for clinical cure and are increasingly understood to represent regrowth of the original infectious strain of *Mtb* in human subjects [43, 45].

A surprising observation in the HIS mouse model of HIV-mediated TB relapse was that regrowth did not appear to coincide with breakdown of contained granulomas as is generally assumed to occur [56]. Instead, lung lesions were observed to mostly resolve following 2 months of TB chemotherapy. Following HIV infection in mice with drug-treated TB, increased mycobacterial regrowth was observed in lung as well as liver prior to redevelopment of organized granulomas. These observations should be noted cautiously pending follow up studies in advanced models such as NHP where the natural history of disease can be followed, and granuloma development and architecture has been more fully characterized. Use of mycobacterial strains carrying a molecular barcode and advanced imaging approaches described in NHP models [57] could be further applied to humanized mice to understand trafficking and redistribution of paucibacillary *Mtb* following TB chemotherapy and regrowth driven by HIV.

HIV replication was also observed to increase in lung and liver of mice with relapsed TB as compared to mice with HIV mono-infection. Interestingly, a marked difference was observed in these tissue compartments despite comparable viral load in the blood [18]. RNA scope analysis further revealed the presence of viral RNA in areas of interstitial and solid lesion inflammation due to *Mtb* including in the T cell zones of lung granulomas. The increased viral replication correlated with increases in several cytokines that activate the HIV LTR (e.g. IL-1β, TNF-α, and IL-6) due to *Mtb* proliferation. An interesting hypothesis supported by these observations, is that localized inflammation at sites of paucibacillary *Mtb* containment could promote proliferation of an HIV reservoir. In return, or potentially simultaneously, the compromise of local immunity by HIV may permit growth and dissemination of *Mtb* away from the localized site. The small animal nature of the HIS mouse is an advantage that could be exploited to investigate these and other mechanisms of microbial synergy. Important caveats of Cornell or Cornell-like models that should be considered are the requirement for drug treatment to generate paucibacillary infection, and reports that suggest rates and kinetics of reactivation can be difficult to reproduce with precision in different groups of animals [55]. The additional variability resulting from the biological or genetic differences in hematopoietic stem cells from different human donors used in HIS mouse generation could further contribute to this challenge. Implementation of experimental indicators (e.g. weight loss or inflammatory biomarkers) of disease progression, rather than time, are likely to be important tools needed to standardize and interpret results in these models.

## Impact of co-infections on deep tissue compartments including reservoirs

Several lines of evidence suggest that plasma viral load, which is used diagnostically to assess viral suppression status, does not always reflect replication in other tissue sites. This question is especially relevant in the setting of co-infections where inflammation and other factors can promote HIV pathogenesis in local tissue sites. Notably, viral shedding in lung lavage of those with concurrent *Mtb* has long been known to be higher than *Mtb*-naïve counterparts despite comparable plasma values [58]. Similarly, HIV co-infection is associated with higher semen, but not plasma, viral loads in men with urethritis due to *N. gonorrhoeae* infection [59]. As previously discussed, both of these clinical disease outcomes have been reproduced in HIV-infected HIS mice that were co-infected with *Mtb* or *N. gonorrhoeae* [19, 20]. We observed similar viral load in plasma of mice infected with the HIV-1 JR-CSF strain, while HIV copies in the lung and liver were markedly increased in mice with *Mtb*. Xu and colleagues similarly demonstrated that vaginal shedding of HIV-1 BaL was increased in mice with *N. gonorrhoeae* co-infection, an outcome also not reflected in the plasma [20].

**Fig. 1:**
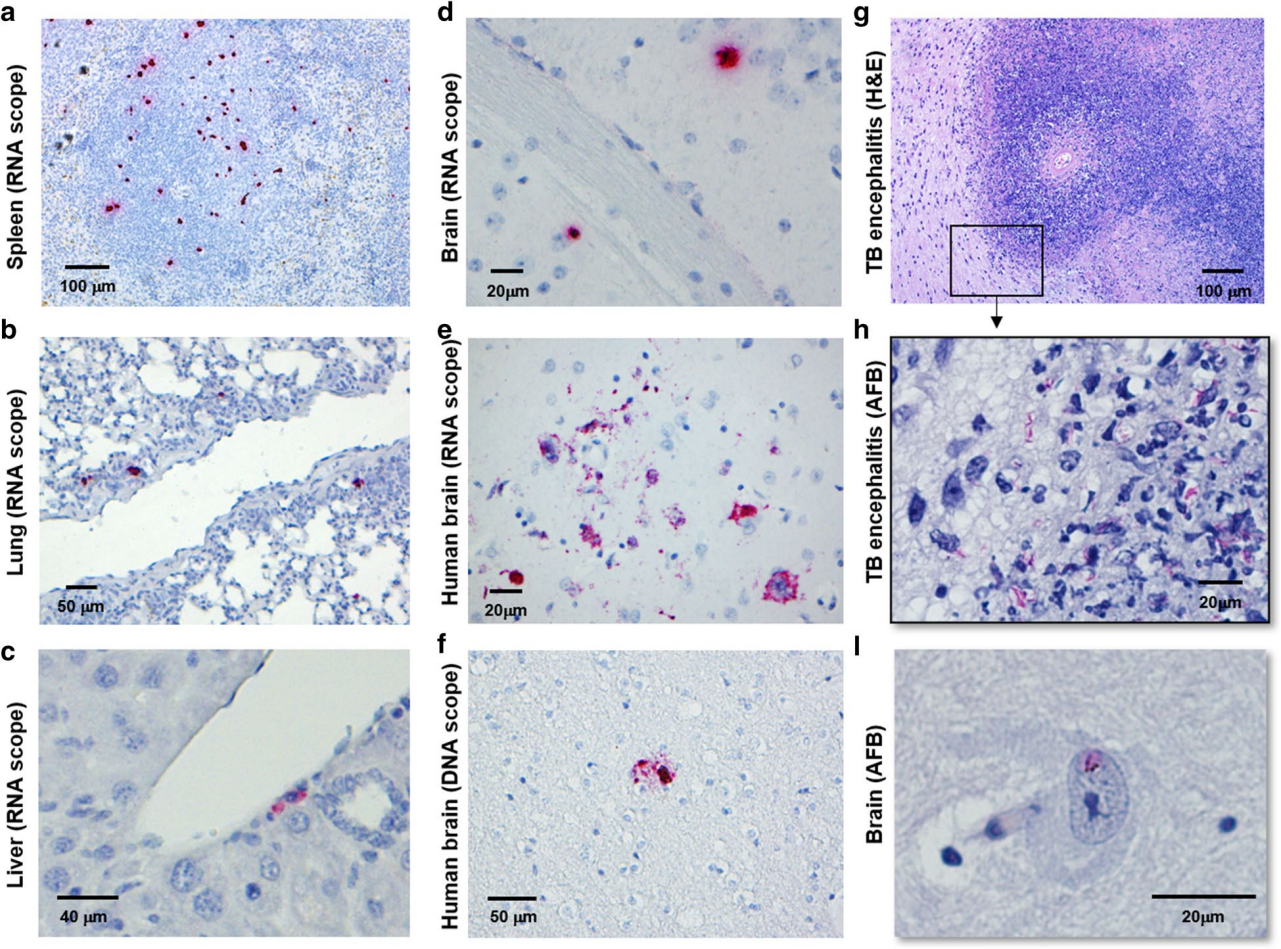
HIV Infection of Multiple Tissue Compartments Supports Potential for Co-infection Investigations in HIS Mice, including in CNS. Results shown in **A**–**D** are from mice that were infected with 2500 TCID_50_ of HIV JR-CSF using an i.v. route and tissue collected after 3 weeks of infection. Autopsy specimens of human brain displaying HIV encephalitis are shown in E and **F** Formalin-fixed and paraffin embedded tissue sections were cut, dewaxed, and viral RNA (**A**–**E**) or DNA (**F**) detected by using in situ hybridization with probes specific to the HIV-1 *gag* gene using RNA Scope® or DNA Scope® (ACD Bio) (**A**–**F**). Staining was visualized with bright field microscopy. Representative images of results from mice used in various studies is shown. Results displayed in **G**–**I** are from HIS BLT mice infected i.n. with 10^2^ CFU of *Mtb* H37Rv for 4 weeks. Shown is development of TB encephalitis as visualized with H&E (**G**) and pockets of AFB (**H**) in the inflammatory lesion. Brains of *Mtb*-infected mice lacking significant neuropathology (**I**) also occasionally presented with a rare AFB (**I**). These results are illustrative of observations from several different studies performed with humanized NSG-BLT mice with mono-infections of HIV or *Mtb*

An important advantage of HIS mouse models is the potential to assess determinants of replication and reservoirs in tissue compartments under experimentally controlled settings. As shown in our on-going studies in Fig. 1A–D, human cells that are actively replicating HIV are found distributed throughout tissues in BLT HIS mice including brain, lung, liver, and spleen. Replication in several tissues including colon, uterus, and vagina have also been described [20] as well as latent infections. This wide distribution of HIV^+^ cells throughout humanized mouse tissues is an important endpoint in the model that enable studies of tissue specific pathogenesis. This facilitates investigation of viral replication and latent reservoirs in difficult to assess compartment such as the CNS, as shown with human brain (Fig. 1E, F) where HIV RNA markings are predominantly observed in microglial cell processes of autopsy specimens from a case of HIV encephalitis.

HIS models should emulate the neurological picture illustrated in human brain (Fig. 1E, F) prior to ART administration, where RNA predominates over DNA. Following ART administration, greater DNA should be observed in brain or other tissues of suppressed HIS mice in order to emulate human disease and demonstrate relevance to viral reservoir modeling. Modeling of TB and HIV dual infections of the CNS are also likely to be feasible based on our preliminary observations. As illustrated in Fig. 1G–I, *Mtb* proliferation and development of TB encephalitis are also observed in the HIS BLT model. These features of the HIS mouse could be further exploited to advance models of co-infection that explore events at tissue sites that are challenging in human subjects or NHP models due to experimental control, tissue availability, or cost.

In the case of HIV and TB, reproducing co-infection in multiple tissue sites such as the brain, lung, spleen and liver as illustrated in Fig. 1, permit investigations that are not feasible otherwise. An important note in this regard is that many of the effects of HIV are mediated by viral proteins and pathogen recognition receptor events of host cells (including mouse) that do not require direct cellular infection. This advantage related to understanding the indirect effects of HIV infection poses a caveat for interpretation of some co-infection outcomes. The small numbers of dysfunctional murine myeloid cells that persist in the NSG and other background strains used for humanized mouse generation [60], for example, could contribute to outcomes of *Mtb* infections. Murine macrophages in cord blood and BLT HIS mouse models have been shown to support mycobacteria replication [34, 35]. Comparisons of humanized and non-humanized cohorts infected with *M. bovis* BCG demonstrate that reconstitution with human macrophages, however, promotes greatly increased bacterial loads [34, 35]. Importantly, human macrophages have been shown to predominate in the inflammatory lesions associated with mycobacterial antigen concentration and recruited human T cells [35].

Experimental control of the human immune cell populations represents an important advantage of the HIS mouse. Reconstitution of human immune populations lacking specific compartments is emerging as an important tool for understanding unique roles of cell types in HIV pathogenesis. The development of the myeloid only model (MoM) or T cell only model (ToM) demonstrated importance of the myeloid cells in HIV persistence [61, 62]. Surgical engraftments of human tissue explants in NSG background mice may further allow interrogation of the mechanisms contributing to in vivo disease, as shown to be feasible for pathogens such as Middle Eastern Respiratory Syndrome Coronavirus, Zika virus, and Nipahvirus [63, 64]. These approaches further permit comparisons of tissue-specific disease outcomes in the presence or absence of circulating human immune cells [63, 64], as shown with the recent demonstration of localized *M. bovis* BCG replication and development of immune responses to cytomegalovirus infection in a lung only model (LoM) [64]. Long term, application of these precision models could be important for understanding the role of specific human immune cells dysfunction due to HIV on the outcomes of co-infections in different tissue compartments.

## Modeling the effects of co-infection on ART outcomes

Treatment of people with both HIV and other infections can be challenging due to the complexity and sometimes lengthy period of combination therapy. ART requires lifelong treatment of anti-retroviral cocktails that may require modifications when combined with other medications for non-communicable and communicable diseases. TB chemotherapy requires treatment with a multi-drug regimen for 6–18 months depending on the relative drug susceptibility or resistance of the *Mtb* isolate. The side effects, complexity, access, and cost of the treatments can lead to compliance issues and treatment failure [65]. Pharmaceutical improvements have greatly reduced drug/drug interactions and toxicity issues that previously contributed to clinical challenges. However, gaps remain that are difficult to address using in vitro systems or mono-infection models. Several issues related to “breaks” in viral suppression previously attributed to poor compliance are understood to have a biological component related to an underlying co-infection [66, 67].

**Fig. 2:**
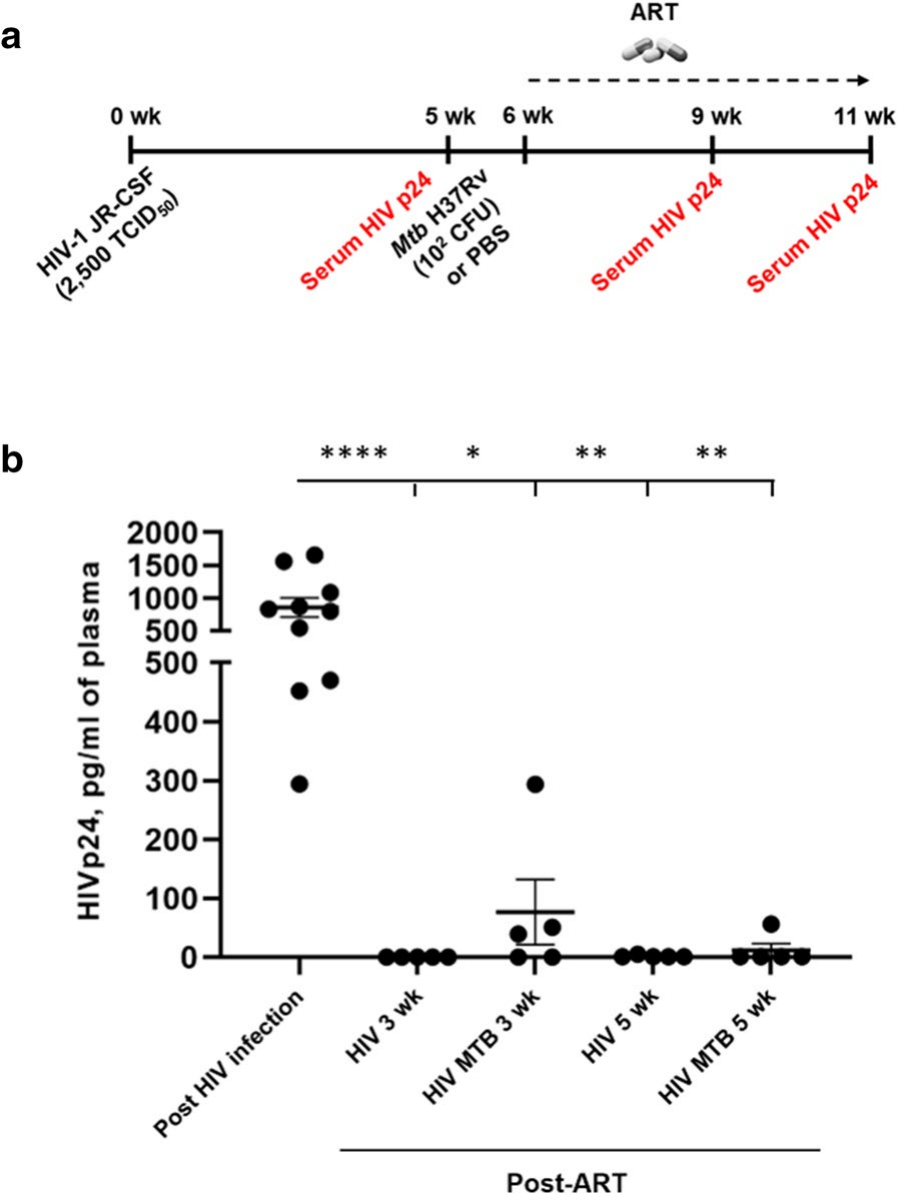
Humanized Mouse Model for Testing Drug Efficacy in Setting of Co-infection.** A** Experimental design for study to test potential for *Mtb* infection to alter ART efficacy in HIS mice with HIV infection. Humanized mice (n = 10) were infected i.v. with 2500 TCID_50_ HIV-1 JR-CSF. At 5 weeks following HIV infection, plasma samples were analyzed for presence of HIVp24 capsid protein. Subsequently, mice were infected with PBS (mock, n = 5) or 10^2^ CFU of *Mtb* H37Rv (n = 5) for another 5 wk. ART was begun in both groups 1 wk post-infection with *Mtb*. **B** Shown is production of HIV p24 capsid protein as detected in serum with a diagnostic ELISA at 5 wk p.i. with HIV, and following 3 and 5 wk of ART treatment. ART was delivered by daily i.p. injection of Emtricitabine (140-200 mg/kg body weight), Raltegravir (56-80 mg/kg body weight), and Tenofovir (146-208 mg/kg body weight). The HIV p24 capsid protein was detected using a commercially available ELISA (Zeptometrix) and results are shown as pg/ml of plasma. Significant differences between post-infection and post-ART treatment are indicated with *p < 0.05, **p < 0.01, and ***p < 0.001, and ****p < 0.0001

Initial drug toxicity and drug/drug interaction evaluations can be done in normal rodent models, however, efficacy testing is relegated to expensive non-human primates or HIS animal models. Among these, the HIS mouse has proven to be reliable, cost effective and reproducible. An additionally important advantage of the HIS mouse is the potential to model co-infection disease and treatment outcomes along with drug interactions and efficacy. In preliminary studies (Fig. 2), we have begun to evaluate the effect of an underlying opportunistic infection on ART efficacy. In this experiment, HIS mice were infected with HIV for several weeks and subsequently infected with *Mtb* or mock-infected shortly before initiation of ART. Animals were then provided a daily regimen of combination ART to evaluate any effects of active *Mtb* infection on viral suppression. A 3 week course of ART led to viral suppression in all *Mtb*-naïve mice consistent with similar reports [68]. In contrast, circulating HIV p24 was still detectable in 3/5 *Mtb*-infected mice at 3 weeks and in 1/5 mice at 5 weeks post-ART initiation. The effects did not reach significance due to variation and small study size. These preliminary findings could suggest that underlying *Mtb* infection impacts efficacy of ART in this model. Expanded studies with larger animal numbers and more rigorous assessments through measurements of time to viral suppression kinetics are needed. Long term, HIS models may have application in the study of OI-driven inflammation, changes in tissue pathogen distribution (e.g. reservoirs), or other mechanisms of microbial synergy that may compromise ART efficacy.

## Humanized mice as models of neurological disease

### Models of HIV-associated neurocognitive disorders (HAND)

Reports using HIS mice as models of neurological disease are increasing in the literature. Potential utility of these models is greatest when attempting to emulate the pathophysiology of neurological diseases that require or involve the human immune system. Infection with HIV-1, which leads to HAND, fits that paradigm well [9, 69]. HIV-1 infection exhibits strict tropism for the human immune system and affects central nervous system (CNS) function adversely. HAND occurs with and without antiretroviral therapy (ART) [70] which creates a puzzling “gap” between the sharp lowering of virus burden in blood plasma versus persistent HAND [71]. There is an analogous “gap” between the neurovirological data and HAND in virally suppressed patients [72]. In patients without ART, CNS dysfunction was strongly linked to replicating HIV and CNS inflammation (HIV encephalitis; HIVE) [73].

In patients given ART, HIVE and CNS viral replication virtually disappear while HAND persists. Thus, neuropathological and neurovirologic substrates of HAND remain essentially unknown, which in turn makes it difficult to emulate the clinical picture in animal models. Attempts to reproduce HIVE pathology in HIS mouse models are interesting, and represent an essential step forward [72]. Disease endpoints that establish clinical relevancy to HAND in patients taking ART remain to be demonstrated. A critical next step is to treat the animal with ART, reverse the neuropathological features of HIVE and then observe residual CNS dysfunction in the animal. Achieving the key next step would emulate the characteristic virological “gaps” that are observed in patients treated with ART.

Some studies using HIS mice have taken steps to replicate aspects of HIV infection of the CNS. The first and most basic hurdle was to produce CNS infection with HIV-1 in the HIS mouse. This has been demonstrated in several reports that utilized a variety of HIS models [74–79]. These models are potentially useful for studies of virus reduction, and in some cases, to study eradication of HIV DNA in the CNS [9]. It is difficult to determine what experimental paradigm of viral eradication HIS models should emulate. Specifically, the types of human inflammatory cells in xenografted mouse models are different than what is found in a native human brain. Many HIS mouse models do not replace murine astrocytes and microglia, and these cells are important components of the pool of residual HIV DNA in the CNS [9]. The proportionate distribution of various cells that can support HIV-1 infection (macrophages, dendritic cells, lymphocytes) in humans also diverges from what is produced in current HIS mouse models. For example, a myeloid cell type of reservoir of HIV-1 is postulated for the human CNS, yet many HIS mouse models involve infection of lymphoid cell populations preferentially. To overcome that, at least one HIS mouse model has been developed that specifically grafts a human myeloid cell population in the rodent [9]. That model focuses on a crucial aspect of CNS HIV infection, that of the myeloid cell reservoir. Other key aspects of viral persistence, such as yolk sac derived microglia and the role of human astrocytes, are not addressed with this model.

The neuropathological changes observed in many HIS mouse models are usually subdued and do not represent the full neuropathological picture of HIV encephalitis (HIVE). Only moderate inflammatory changes have been produced [13, 80, 81]. By comparison, neurosurgical xenograft type models of HIVE in which the brain is directly injected with HIV-1 infected human cells (SCID mice) often produce more intense and “accurate” HIVE type inflammatory changes versus what is produced in HIS mice [79]. A key limitation in development of suitable HIS mouse models of HAND is that the grafting does not replace rodent microglial cells, which function as the animal’s resident histiocytic pool in the CNS. There is potential however, as effective grafting of human microglia was recently accomplished in a humanized bone marrow chimera in which human interleukin-34 under the control of the cytomegalovirus promoter drove brain reconstitution of human stem cell derived peripheral macrophages to differentiate into human microglial-like cells in the mouse brain [82]. These microglial-like cells were capable of hosting in vivo infection with a myeloid trophic viral isolate. Although not yet widely available, these HIS mice represent a major advance, because microglial cells are a main source of replicating HIV in the CNS and they probably contribute HIV DNA to the latent CNS pool in virally suppressed patients.

Engineering rodent models of CNS impairment that faithfully emulate HIV infected patients with viral suppression appears to be within sight, but many challenges remain. Human astrocytes are infected with HIV-1 and may contribute to the size of the latent HIV DNA pool in the CNS. Current HIS mouse models may be less suitable to study that aspect of HIV latency. As well, the regional distribution of infected human cells within the CNS of HIS mouse models might differ from the CNS of people living with HIV infection. High levels of HIV-1 replication in the human brain tend to occur in deep brain structures such as the basal ganglia and thalamus. The mapping of the latent (not replicating) pool of HIV-1 also needs to be elucidated in these models for correlation with human CNS infection. Comparative neurovirology between human and rodent infections is an important aspect with implications regarding the elucidation of circuit level changes in CNS function in patients with HAND. Perhaps most vexing of all is that populations of HIV infected patients harbor comorbidities with higher prevalence than noninfected populations, particularly including substance abuse and chronic exposure to therapeutic drugs with potentially adverse effects in the CNS. These ongoing complexities suggest that specific HIS mouse models may be required to emulate key aspects of human CNS infection.

Further development of HIS mouse models should take the step forward of emulating the CNS inflammatory picture of HIVE, with CNS dysfunction when replication is not suppressed. A key subsequent step involves treating the HIV-infected animals with ART to prevent HIVE and reduce the intensity of CNS dysfunction, while not eliminating it, as observed in ART treated patients. Virally suppressed HIS mice should contain a pool of latent HIV DNA that is comparable to what is observed in human brain specimens including its size, its responsiveness to latency reversing agents, and its neuroanatomical distribution. As well, the HIS mouse response to psychological testing should correspond to systemic anomalies correlated with HAND, such as the association with bacterial gut translocation [83], systemic endothelial dysfunction and systemic virology (63). Given the complexities involved it may not be possible for a single HIS mouse model to faithfully emulate the essential pathophysiology of HAND.

### HIV co-infections of the CNS

Prior to the ART era, CNS forms of several infections such as cryptococcal meningitis, cerebral toxoplasmosis, and tuberculous meningitis were associated with severe clinical outcomes [84]. Neuro-TB also continues to be an important health concern in those with HIV, especially in children [85]. Models of neurological infection with mycobacteria have been developed in guinea pig, rabbits, and standard mouse inbred strains [86]. These models have application to understanding several different aspects of mycobacterial infection and drug penetration. To date, systems that permit investigations of how HIV compromises the CNS immunity to co-infections, or how co-infections may contribute to HAND or maintenance of CNS reservoirs, are lacking.

Various humanized mouse models generated with human leukocytes or stem cells have been used over the past two decades to understand HIV infections in the CNS [87]. To date, however, the potential to investigate the outcomes of co-infections has been limited by lack of studies that demonstrate productive infections of human cells with important co-infections such as *Mtb*. Our previously unpublished work shown in Fig. 1 provides preliminary support for the potential to investigate HIV-associated neurological *Mtb* outcomes. In a limited survey to date, we have occasionally observed acid-fast bacilli (AFB) in the brains of humanized mice after several weeks of *Mtb* infection (Fig. 1I). In animals with high bacterial burden in the lungs and sites of dissemination (e.g. liver), we have also observed development of TB encephalitis (Fig. 1G) associated with areas of *Mtb* proliferation (Fig. 1H).

The CNS is also a well-established tissue reservoir for HIV that is poorly understood and is currently the subject of intensive investigation. A contribution of co-infections is suspected, based on associations, although empirical evidence is lacking. Important questions that could be addressed experimentally in the HIS model include: whether opportunistic infections (OI) increase the CNS viral load or reservoir size; if systemic or tissue compartment inflammation due to OIs promotes HIV CNS outcomes; whether OIs contribute to HIV Associated Neurological Dysfunctions; and what are the mechanistic bases for increased rates or more severe CNS outcomes of OI in those with HIV? Demonstrations that important endpoints of CNS co-infections could be realized in the HIS mouse models would also support use in translational studies to test clinical interventions.

## Humanized mouse models of HIV/Hepatitis virus co-infection

Due to their similar routes of transmission, it is increasingly common for PWH to have a hepatitis virus co-infection. Of the current 39 million PWH [1], 10% are estimated to be co-infected with Hepatitis B Virus (HBV) [88] and 4–12% co-infected with Hepatitis C Virus (HCV) [89], yielding anywhere from 2 to 12 million potential co-infections worldwide.

HIV/HBV co-infection exacerbates morbidity and mortality of both viruses, with death rates of co-infected individuals being 8 times that of HIV infection and nearly 20 times greater than HBV infection [90]. Much of this increased mortality springs from a five-fold greater risk of hepatocellular carcinoma (HCC) in HBV infected patients with HIV [6]. Similarly, HCV-mediated liver damage, including fibrosis [91], cirrhosis and HCC [7], is accelerated in individuals co-infected with HIV. Incidence of cirrhosis was found to be tripled in co-infected subjects as compared to those with HCV infection alone. While the mechanisms of this increased pathogenesis are not well understood, possible causes include loss of T cell function, increased levels of fibrosis-inducing TGFβ, and HIV infection of hepatic stellate cells, among others [92].

Much of the difficulty of studying these co-infections stems from the challenges to establishing animal models of infection. The human-restricted tropism of all three viruses has proven to be nearly insurmountable to gaining insights into single or dual infections. A number of potential models described below have been developed in attempts to overcome this obstacle.

### AFC8-hu-HSC/Hep model

AFC8 mice were originally created to allow for human hepatocyte engraftment into mice to facilitate the study of HCV pathogenesis. A fusion protein consisting of KF506-binding protein (KFBP) and caspase 8 was knocked-in to Balb/c Rag2^−/−^ IL2Rg^−/−^ mice under the control of the albumin promoter, allowing for the inducible destruction of mouse hepatocytes [22]. Neonatal KFBP knock-in mice are irradiated, and human hepatocytes are injected into the mouse liver. Destruction of murine hepatocytes is initiated, and dying mouse hepatocytes are replaced by expanding human cells. This model was expanded into the AFC8-hu-HSC/Hep model by co-injecting human fetal-liver derived CD34^+^ hematopoietic stem cells (HSC) into the irradiated neonatal mouse liver along with the human hepatocytes [93]. The engrafted HSCs then repopulated the immune system with human lineage lymphocytes, enabling the study of the immune response to HCV, along with viral life cycle and pathogenesis. This model should be capable of supporting HIV infection, as well as hepatitis virus infection.

### FNRG models

Other liver xenograft models could be similarly adapted to HIV/hepatitis virus co-infection. The FNRG mouse is a NOD/*rag1*^*−/−*^*/il2rg*^*−/−*^ background with a mutation that disables the *fah* gene, which encodes fumarylacetoacetate hydrolase [94]. These mice also exhibit hepatocyte toxicity unless fed a diet lacking tyrosine and phenylalanine, and supplemented with the drug nitisinone, 2-(2-nitro-4-trifluoro-methylbenzoyl)-1,3-cyclohexanedione (NTBC). This model has also been immune reconstituted with human leukocytes (FNRG-hu-HSC/Hep) and used successfully to examine immune responses to hepatitis virus infections [95, 96].

### HIS/HUHEP model

A similar approach was based upon Alb-uPA/SCID (uPA) mice. In the uPA model, the urokinase-type plasminogen activator was placed under the control of the albumin promoter. The accumulation of plasminogen activator results in mouse hepatocyte death, which are subsequently replaced by engrafted human hepatocytes. Strick-Marchand et al. established a Balb/c Rag2^−/−^ IL2Rγ^−/−^ NOD.*sirpa* uPA^tg/tg^ (BRGS-uPA) strain that carries the same hepatocyte-lethal phenotype, but is better able to support establishment of a human immune system due to the expression of human SIRPα [97]. These mice were engrafted with both human hepatocytes and fetal-liver derived HSCs for the study of HBV infection and the immune response.

### TK-NOG model

The TK-NOG mouse [98] a NOD/SCID/IL2Rγ-knockout mouse which bears a thymidine kinase gene under control of an albumin promoter, also undergoes a selective hepatocyte toxicity, accepts human hepatocytes and is capable of reconstituting a human immune system (TK-NOG-hu-HSC/Hep). This model was recently adapted to HIV infection [99] to examine the effects of HIV on hepatocyte function. Dagur et al*.* reconstituted TK-NOG mice with human hepatocytes and CD34^+^ HSCs. The resulting mice showed functional human hepatocytes secreting serum albumin and human CD45^+^ lymphocyte populations consisting of CD4^+^ and CD8^+^ T cells and B cells. These humanized liver and immune system mice were subsequently infected them with HIV. Decreases in CD4:CD8 ratios and serum albumin levels were detected in at 5 weeks post-infection. This successful demonstration of HIV infection in this model makes the TK-NOG-hu-HSC /Hep model a leading candidate for the study of HIV/Hepatitis virus co-infection.

Hepatic replacement models overcome the challenge of infecting mice with viruses that have a specific tropism for human cells, such as HIV, HBV and HCV. While none of the above models, to our knowledge, has yet been used to examine HIV/HBV or HIV/HCV co-infection, they should all be suitable for that purpose. Models that can establish a human immune system from CD34^+^ cord blood HSCs would be particularly useful, given the regional restrictions on use of human fetal tissues. Such HIS-huHEP mouse models would have significant advantages over other potential animal models. SHIV/HCV co-infection of chimpanzees (*Pan troglodytes*), for example, should be feasible, but would be prohibitively expensive and challenging from a regulatory standpoint in many parts of the world. HIS-huHEP mice are cheaper, more easily housed and enjoy a far greater breadth of available reagents. Other potential animal models lack the ability to study genuine human-infectious pathogens in the context of a human immune response. HIS-huHEP mice afford the opportunity to study native HIV, HBV and HCV, in the appropriate host cells, in the context of a relevant human immune response. Studies of liver fibrosis, immune cell dysfunction, HIV viral reservoirs and a host of other pathologies of co-infection are all uniquely possible in these systems.

These models may have some shortcomings, however, particularly with regard to vaccine testing and development, owing to the incomplete reconstitution of the B cell compartment in most HIS mouse models. Also of significant concern is the matter of immune cell compatibility between the tissue donors. Graft vs. host disease is an ongoing issue in HIS mice, with the reconstituted immune system attacking the host animal as non-self. HIS-huHEP mice will likely have similar issues, possibly compounded by graft vs graft disease, with the reconstituted immune system reacting to both the host animal and the engrafted liver if they are not sourced from the same donor.

### HIL model

More recently, a simpler model has been shown to be useful in the study of HCV infection. In the HIL model [100], the injection of human fetal hepatic progenitor cells into NSG mice was demonstrated to yield both human hepatocytes and HSCs, resulting in human liver engraftment and the establishment of a matched human immune system. Furthermore, virus-specific lymphocytes and responsiveness to drug treatment were observed.

The HIL model has significant advantages over other HIS-huHEP models. It is much simpler to establish, as it does not require murine hepatocyte ablation. Hepatic progenitor cells have been shown to engraft directly into the mouse liver [101]. It also eliminates the possibility of graft vs graft disease, as the resultant human hepatocytes and immune cells arise for the same progenitors. One potential shortcoming, however, is the use of fetal tissue. This will unfortunately limit the models use in areas where use of fetal tissue is prohibited.

### HLA transgenic models

Another set of models that may be useful in the study of HIV/HBV or HIV/HCV coinfection are those that carry human leukocyte antigen (HLA) genes. HLA transgenic (HLA-tg) mice were developed to study the nature of antigen recognition by T cell receptors in the context of HLA molecules [102], and later adapted to the study of immunogenic epitopes of human-tropic pathogens [103]. HLA-tg mice have been produced that carry numerous common HLA genes, including A2, A3, A11, B65, DR1, DR4, and DR7 families, among others, that have been used to study the immune response to numerous viruses including HIV, HBV, HCV, Cytomegalovirus and poxviruses [103–108].

This model was further adapted to the study of HIV by crossing HLA-tg mice with NSG mice (described above), resulting in HLA-tg/NSG mice [109]. These mice could then be reconstituted with a human immune system by injection of CD34^+^ HSCs (HLA-tg/NSG-HIS), hopefully overcoming the limited T cell-dependent B cell activation and cytotoxic T cell killing often observed in reconstituted NSG mice.

The HLA-tg/NSG-HIS model could be further applied to the study of HIV/HBV or HIV/HCV co-infection. Such models can be chronically infected with HIV, and either pulsed with HLA-specific hepatitis virus antigens or hydrodynamically injected with DNA encoding hepatitis virus proteins. Hydrodynamic injection is a method in which HCV cDNA or an HBV encoding plasmid is injected intravenously in PBS equivalent to ~ 10% of the animals’ weight in less than 10 s [110]. This rapid infusion of liquid causes body tissues, particularly the liver, to uptake DNA and begin expression of the encoded proteins, yielding a surrogate infection in a non-permissible tissue. While the transfected cells do not produce progeny virus, they do produce all encoded viral proteins and elicit many effects of genuine infection.

HIV-infected, viral protein producing, HLA-tg/NSG-HIS mice could have utility in a number of areas. They would be particularly useful in studying the immune reaction to HBV or HCV proteins in the context of HIV infection, as all the immune dysfunction of HIV infection would be present. Similarly, candidate hepatitis vaccines intended for use in PWH could undergo initial animal testing in HLA-tg/NSG-HIS mice. The effects of HBV or HCV infection on the establishment and maintenance of the latent HIV reservoir could also be examined is this model, as transfected hepatocytes replicate most aspects of chronic viral infection, including long-term inflammation.

The HLA-tg/NSG-HIS model would be of more limited utility in studying the mechanisms by which HIV infection exacerbates viral hepatitis, as this model does not replace murine with human hepatocytes. While most viral proteins are expressed and most aspects of infection are reproduced, the lack of viral replication, cell-to-cell spread and dsRNA in infected cells are formidable obstacles to the study of hepatic disease. Such investigations would be best left to the hepatic replacement models described above.

## Co-infections of the reproductive tract

A bidirectional relationship between HIV and several sexually transmitted infections (STI) is well established. Beyond the common route of transmission and overlapping epidemiological factors, HIV increases the susceptibility to, and disease outcome of, many STIs [5]. Similarly, several STIs including *Chlamydia trichomonas*, *N. gonorrhoeae*, and Herpes simplex-2 also increase the risk for HIV infection through reductions in barrier effectiveness, activation of HIV co-receptors, and inflammation [5]. Clinical presentations in PWH that have co-infection with *Neisseria gonorrhoeae,* however*,* suggest microbial synergy may also occur [59, 111]. Increased viral load in semen of those with *N*. *gonorrhoeae* co-infection has been demonstrated, including after viral suppression with ART [59]. The mechanistic basis for co-infection synergy among HIV and important STIs is poorly understood to date. The human tropism for both pathogens has previously limited the availability of animal models to study co-infection.

An important advance for modeling outcomes of HIV in the reproductive tract has been the demonstration of rectal and vaginal mucosal infections in the HIS mouse [12, 16, 61]. These innovations facilitated development of co-infection models for important sexually transmitted diseases in the HIS mouse, such as the recently described model of HIV and *N*. *gonorrhoeae* co-infection [20]. Xu and colleagues demonstrated that the gut and reproductive tract of NSG mice engrafted with CD34^+^ stem cells are well populated with human immune cells. Importantly, human T cells including abundant CD4^+^CCR5^+^ T lymphocytes that support productive HIV replication were described. Viral shedding could be easily detected in vaginal washes of mice that had been infected with HIV by an i.p. route. Co-infection with *N*. *gonorrhoeae* was observed to increase the time to establishment of HIV proliferation in the reproductive tract, and to markedly increase viral shedding in vaginal washes, compared to HIS mice infected with HIV only. The description of this small animal model of HIV and *N*. *gonorrhoeae* supports further investigations of mechanisms driving microbial synergy and provides a translational platform to test microbicides and other clinical interventions. Long term, the demonstration that the HIS mouse repopulates these important human immune components and supports HIV infection in the gut and reproductive tract further supports use of the CD34^+^ cord blood models for co-infection studies when use of the BLT mouse is limited.

Development of HIS models of HIV co-infections with other important pathogens which are transmitted sexually is feasible. Productive infection with herpes simplex virus 2 (HSV-2) and development of human immune responses to the virus was previously demonstrated in an HIS mouse developed using cord blood stem cells [112]. Once developed, a dual infection model of HIV and HSV-2 would have significant utility for basic discovery related to microbial synergy and development of clinical interventions in co-infected or at risk populations.

## Conclusions

Improvements in engraftment of human immune compartments in xenochimeric mice continue to support expanded applications of these models to HIV research. These advances have now permitted successful exploration of HIV disease in multiple compartments, including co-infections of the respiratory system and reproductive tract, that reproduce important features of the respective human disease. Recent advances in hepatocyte regeneration have further enabled development of hepatitis virus models in HIS mice that could additionally support modeling of HIV co-infections with these important causes of cancer. Many caveats to the use of HIS mice to study HIV as well as HIV associated co-infections remain, most notably the xenochimeric immunity and physiology. Care must be taken to define the research questions as appropriate to the model advantages and limitations. Validation of the outcomes using human specimens and human clinical research is also central to ensuring advances have translational relevance. Long term, HIS mouse models of HIV-associated co-infections are likely to yield key insights into mechanisms of microbial synergies and provide an informative and economical platform for testing clinical interventions.

## Data Availability

All data generated or analysed during this study are included in this published article [and its supplementary information files].
